# Deep-sea corals provide new insight into the ecology, evolution, and the role of plastids in widespread apicomplexan symbionts of anthozoans

**DOI:** 10.1186/s40168-020-00798-w

**Published:** 2020-03-12

**Authors:** Samuel A. Vohsen, Kaitlin E. Anderson, Andrea M. Gade, Harald R. Gruber-Vodicka, Richard P. Dannenberg, Eslam O. Osman, Nicole Dubilier, Charles R. Fisher, Iliana B. Baums

**Affiliations:** 1 Biology Department, Pennsylvania State University, University Park, PA USA; 2 Department of Symbiosis, Max Planck Institute for Marine Microbiology, Bremen, Germany; 3 Epic, Madison, WI USA; 4 Marine Biology Department, Faculty of Science, Al Azhar University, Cairo, Egypt

**Keywords:** Deep-sea, Corals, Apicomplexans, LIPOR, Symbiont

## Abstract

**Background:**

Apicomplexans are the causative agents of major human diseases such as malaria and toxoplasmosis. A novel group of apicomplexans, recently named corallicolids, have been detected in corals inhabiting tropical shallow reefs. These apicomplexans may represent a transitional lifestyle between free-living phototrophs and obligate parasites. To shed light on the evolutionary history of apicomplexans and to investigate their ecology in association with corals, we screened scleractinians, antipatharians, alcyonaceans, and zoantharians from shallow, mesophotic, and deep-sea communities. We detected corallicolid plastids using 16S metabarcoding, sequenced the nuclear 18S rRNA gene of corallicolids from selected samples, assembled and annotated the plastid and mitochondrial genomes from a corallicolid that associates with a deep-sea coral, and screened the metagenomes of four coral species for corallicolids.

**Results:**

We detected 23 corallicolid plastotypes that were associated with 14 coral species from three orders and depths down to 1400 m. Individual plastotypes were restricted to coral hosts within a single depth zone and within a single taxonomic order of corals. Some clusters of closely related corallicolids were revealed that associated with closely related coral species. However, the presence of divergent corallicolid lineages that associated with similar coral species and depths suggests that corallicolid/coral relations are flexible over evolutionary timescales and that a large diversity of apicomplexans may remain undiscovered. The corallicolid plastid genome from a deep-sea coral contained four genes involved in chlorophyll biosynthesis: the three genes of the LIPOR complex and *acsF*.

**Conclusions:**

The presence of corallicolid apicomplexans in corals below the photic zone demonstrates that they are not restricted to shallow-water reefs and are more general anthozoan symbionts. The presence of LIPOR genes in the deep-sea corallicolid precludes a role involving photosynthesis and suggests they may be involved in a different function. Thus, these genes may represent another set of genetic tools whose function was adapted from photosynthesis as the ancestors of apicomplexans evolved towards parasitic lifestyles.

Video abstract

## Background

Colonial corals at all ocean depths [1] form diverse [2–4] and economically [5] valuable communities that face a variety of threats, including increasing ocean temperatures [6], ocean acidification [7, 8], nutrient runoff [9], disease [6], and oil and gas extraction and production activities [10, 11]. These stressors have prompted the investigation of the role of microbes in the physiology of the coral holobiont [12, 13] and how these microbes may mitigate or exacerbate these stressors [14]. The best-studied coral symbionts are the widespread, eukaryotic, photosynthetic algae in the family Symbiodiniaceae [15]. Recently, two additional alveolate, phototrophic coral symbionts were discovered, which are both close relatives of apicomplexans: *Chromera velia* [16, 17] and *Vitrella brassicaformis* [18]. These organisms attracted attention not only for their potential role as auxiliary phototrophic symbionts but also for their phylogenetic position basal to apicomplexans. They have been postulated to represent a transitional lifestyle between free-living phototrophs and obligate parasites and may shed light on the evolutionary history of important parasites of humans, such as the apicomplexans *Plasmodium* spp. and *Toxoplasma gondii* [16, 19].

In addition to these phototrophic organisms, evidence of other widespread apicomplexan-related symbionts present in corals is mounting. First, a coccidian apicomplexan, *Gemmocystis cylindrus*, was described by transmission electron microscopy in the mesenterial filaments of multiple Caribbean corals [20]. Later, nuclear encoded 18S rRNA gene sequences of apicomplexan relatives were found in corals in the Caribbean and Great Barrier Reef and named “genotype N” [21, 22]. Genotype N was later shown to cluster alongside coccidians within Apicomplexa [19]. Similarly, 16S rRNA gene sequences from the plastid of apicomplexan relatives appeared in bacterial surveys of corals pantropically [23–25]. These plastid sequences form a highly diverse group that clusters basally to apicomplexans and were thus named apicomplexan-related lineage V (ARL-V) [24].

**Fig. 1:**
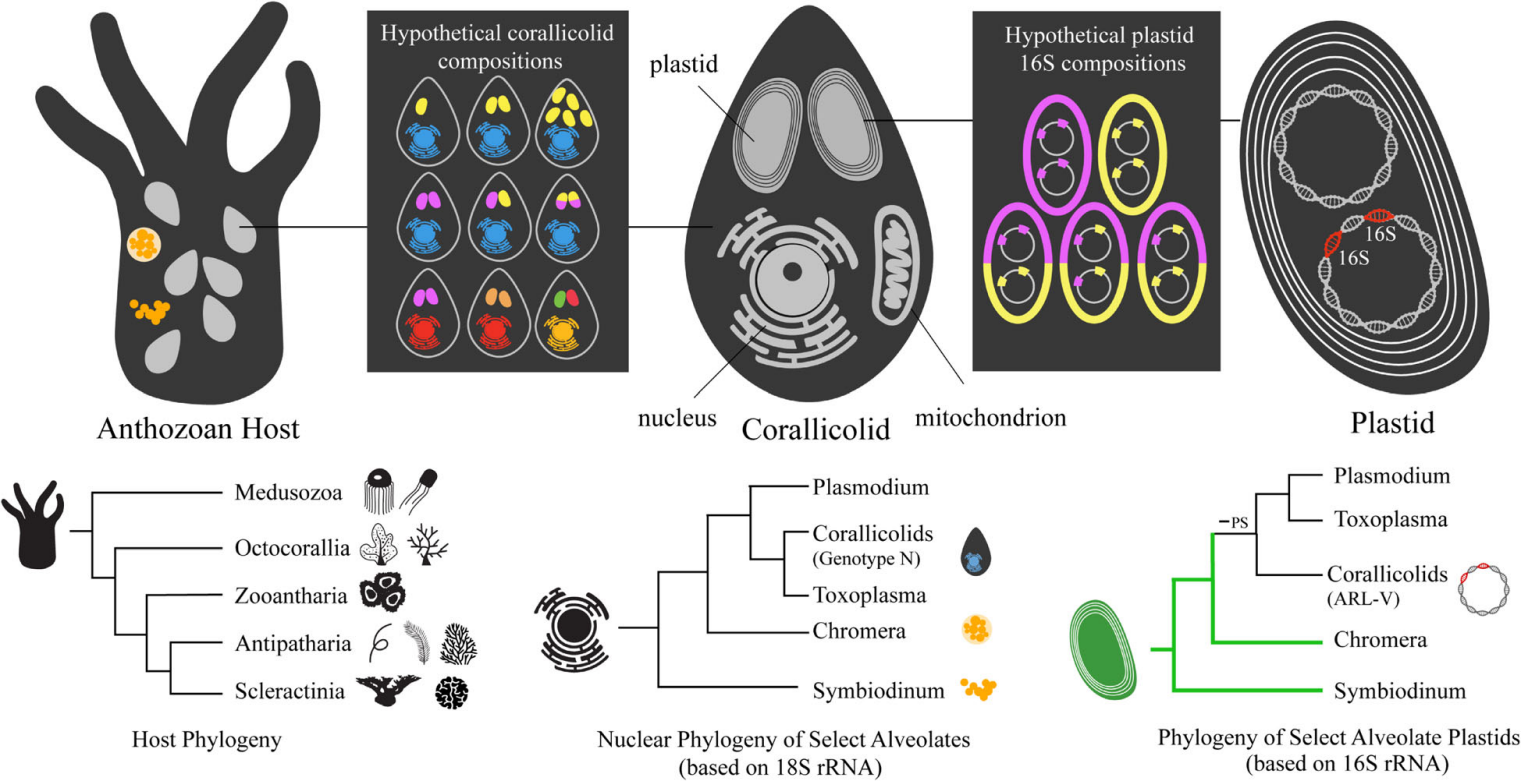
The genomic compartments of corallicolid apicomplexans, the possible diversity in plastid and nuclear compositions, and the phylogeny of host and corallicolid genomic compartments. -PS denotes the loss of photosynthesis. Cnidarian phylogeny based on [26, 27]. Art by Justin Wheeler

It has been hypothesized that some or all of these sequences assigned to genotype N and ARL-V arise from the same organism despite the differences in phylogeny between these nuclear and plastid-encoded genes (Fig. 1) [22]. Recently, co-localized signals were observed in the mesenterial filaments of *Rhodactis* sp. using probes designed for cytosolic and plastid ribosomes of genotype N and ARL-V, respectively, supporting the hypothesis. These organisms were named “corallicolids” [28]. The localization of signals from ARL-V, genotype-N, and *G*. *cylindrus* within the tissue of corals suggests that they are endosymbionts, yet their role in the holobiont remains unclear.

In contrast to some apicomplexans like the parasite *Plasmodium* spp., neither genotype N nor ARL-V are associated with any pathology and are commonly found in association with apparently healthy corals [23, 29, 30]. In addition, genotype N and ARL-V occur at high prevalence, having been detected in over 80% of adult colonies in some scleractinian species [29, 30]. Further, genotype N occurs in a high percentage of the planulae of some brooding coral species suggesting vertical transmission in these species and an association that persists throughout the life cycle of the coral [29, 30].

The role of ARL-V was thought to be phototrophic since it appears basal to apicomplexans and was initially reported to decrease in abundance with depth [23]. However, it was recently shown that the plastid genome of a corallicolid isolated from *Rhodactis* sp. lacks the full genomic repertoire necessary to conduct photosynthesis [28]. The plastid genome did, however, include genes involved in chlorophyll biosynthesis suggesting that corallicolids may produce chlorophyll or a derivative of chlorophyll that interacts with light or is involved in heme regulation [28]. Whether corallicolid apicomplexans are parasites with minimal impact on host fitness, commensals, or mutualists is still unclear.

Most work on corallicolids has focused on shallow-water coral species from reefs in tropical latitudes [19–24, 28–31]. These apicomplexans have only been detected in a few cold-water coral species [25]. However, the majority of known coral species live deeper than 50 m [1], and the communities they form have a much wider range than tropical corals, occurring along most continental margins from the Arctic to the Antarctic [32, 33]. Here, we investigate the occurrence of corallicolids in deep-sea and mesophotic scleractinians, antipatharians, zoantharians, and alcyonaceans from the Gulf of Mexico, ranging in depth from 60 to 2224 m, with comparison to shallow-water corals from the Florida Keys and Curaçao. We also investigate phylogenetic patterns among corallicolids across host phylogeny, geography, and depth using plastid and nuclear marker sequences. Finally, we probe its role in deep-sea corals by analysis of the plastid genome sequence of a corallicolid that associates with the globally distributed deep-sea antipatharian *Leiopathes glaberrima* [Esper 1788] [34–36].

## Results

### Plastid and mitochondrial genomes of corallicolids

**Fig. 2:**
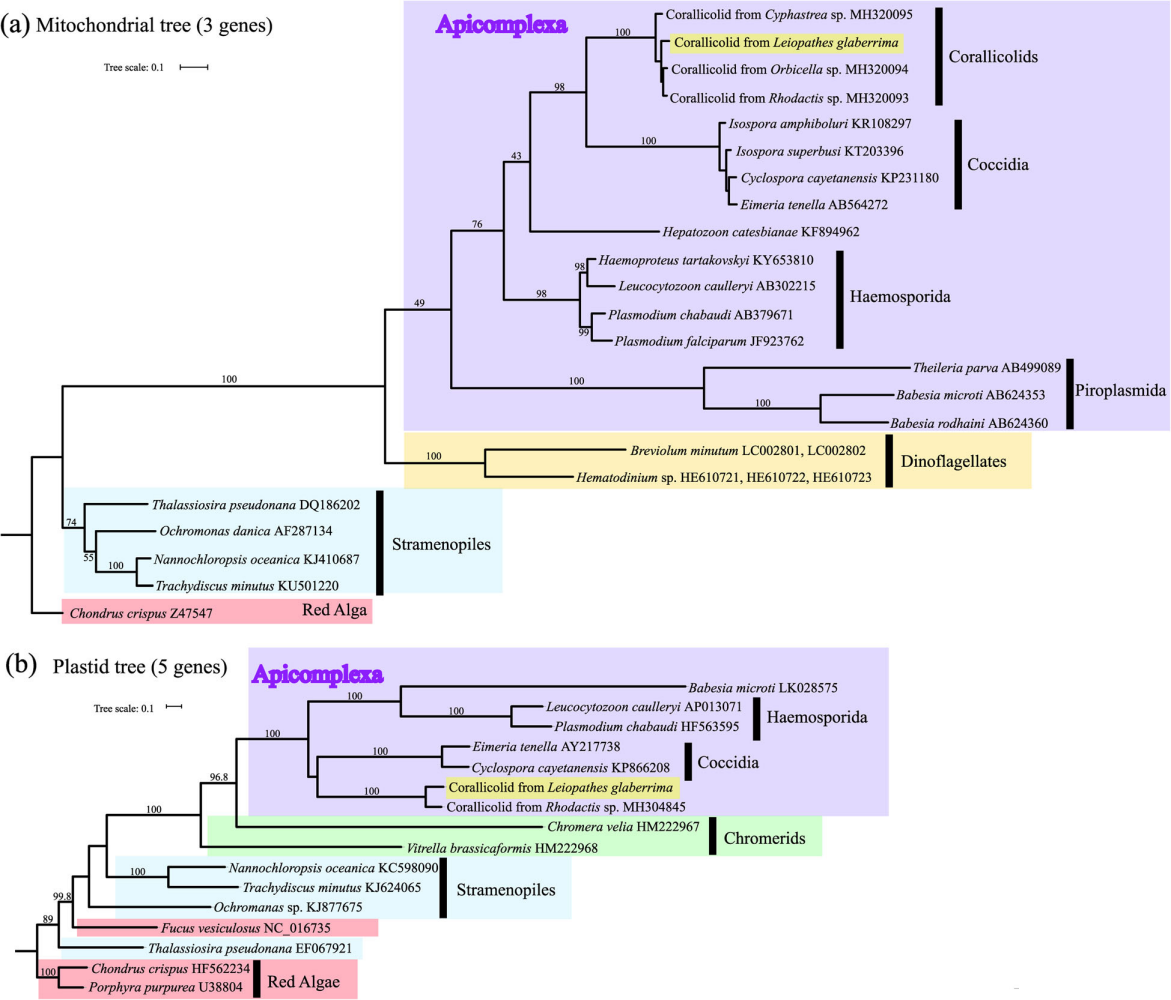
Concatenated tree of **a** mitochondrial genes: *cytb*, *cox1*, and *cox3* and **b** plastid genes: *clpC*, *rpoB*, *rpoC1*, *rpoC2*, and *tufA.* Sequences generated in this study are highlighted in yellow

A 6300 bp mitogenome of apicomplexan origin was assembled from a colony of *Leiopathes glaberrima* collected from a depth of 450 m at site Viosca Knoll (VK) 826. Site names follow the Bureau of Ocean Energy Management’s lease block designations which consist of a two-letter region abbreviation followed by three digits. Like other apicomplexans, this mitogenome contained multiple rRNA fragments and only three complete genes: *cox1*, *cox3*, and *cytb* [37, 38]. A phylogenetic tree placed this apicomplexan with other corallicolids alongside coccidians using amino acid sequences of these three genes (Fig. 2).

From this same metagenomic library, a 45,543 bp plastid genome corresponding to ARL-V was assembled that was composed of two scaffolds. This plastid genome contained four genes involved in chlorophyll biosynthesis that are not present in the apicoplast genome of any other, non-corallicolid, apicomplexan [19, 28]. Three of these genes compose the light independent protochlorophyllide oxidoreductase (LIPOR) complex (*chlL*, *chlN*, *chlB*), and the fourth is aerobic magnesium-protoporphyrin IX monomethyl ester cyclase (*acsF*). A phylogenetic tree of the five longest protein-coding plastid genes also showed that the corallicolid in *Leiopathes* clustered alongside coccidian apicomplexans (Fig. 2).

### Presence of corallicolids in metagenomes

**Fig. 3:**
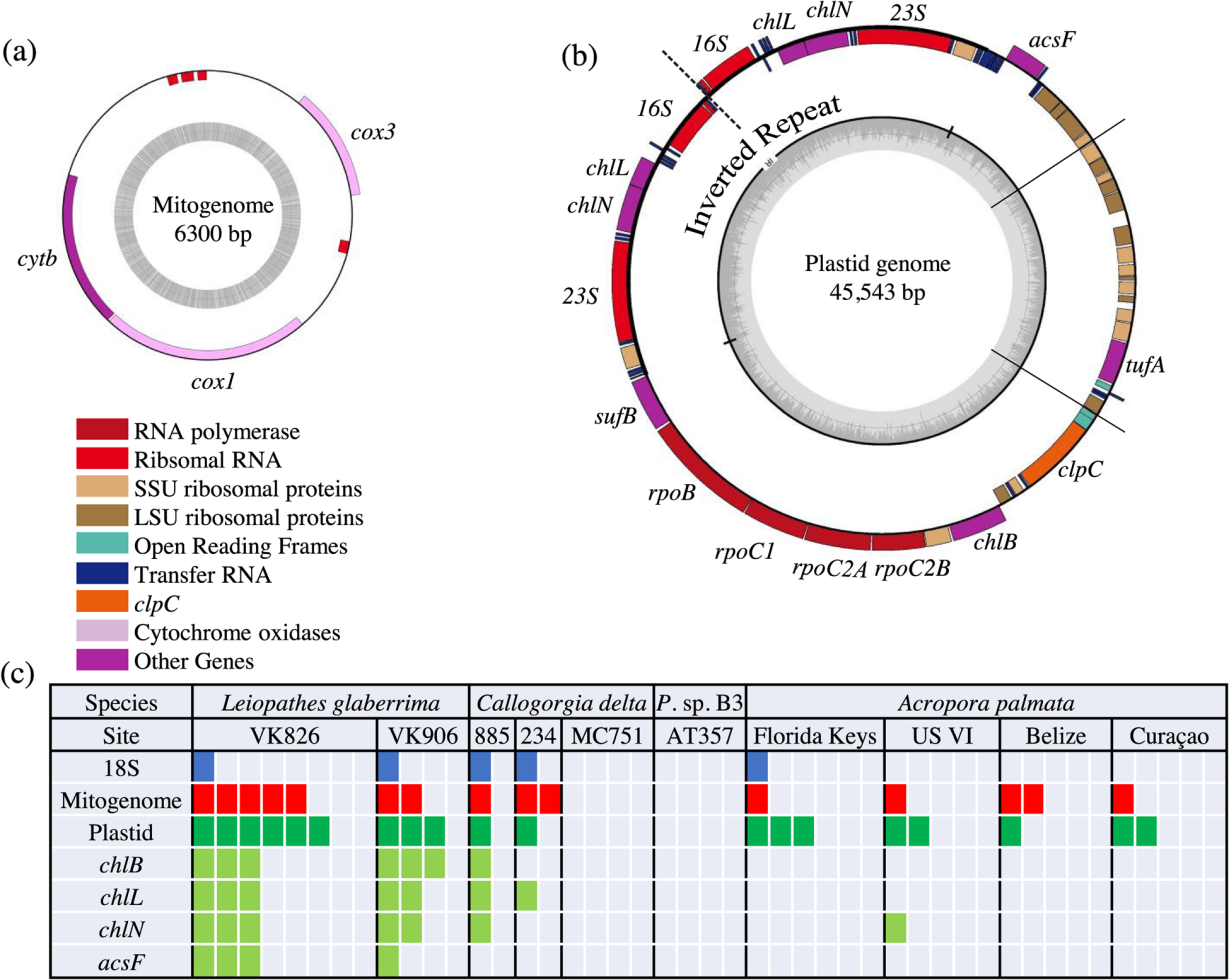
Annotation of the mitogenome (**a**) and plastid genome (**b**) of a corallicolid from *Leiopathes glaberrima.* Radial lines separate two scaffolds of the plastid genome. **c** Detection of corallicolids in the metagenomes of various coral species using mitogenomes, plastid genomes, and selected genes. Each column represents a separate coral colony. *US VI* U.S. Virgin Islands

Corallicolid 18S, mitogenomes, and/or plastid genomes were detected in the metagenomes of *L*. *glaberrima* (9/12), *Callogorgia delta* (3/8), and *Acropora palmata* (9/21) but not in any *Paramuricea* sp. type B3 (*n* = 4) (Fig. 3). Corallicolids were detected in multiple colonies of *A*. *palmata* in all four sampled regions spanning the Caribbean: Florida Keys, US Virgin Islands, Belize, and Curaçao. Corallicolids were only detected in *C*. *delta* colonies from lease blocks Mississippi Canyon (MC) 885 (1/2) and Green Canyon (GC) 234 (2/2) and were not detected in any colony from MC751 (*n* = 4). Finally, LIPOR genes and/or *acsF* of corallicolid origin were detected in two deep-sea coral species: *L*. *glaberrima* (6/12) and *C*. *delta* (2/8), as well as the shallow-living *A*. *palmata* (1/21).

### 16S screening for plastids across a wide diversity of corals

**Fig. 4:**
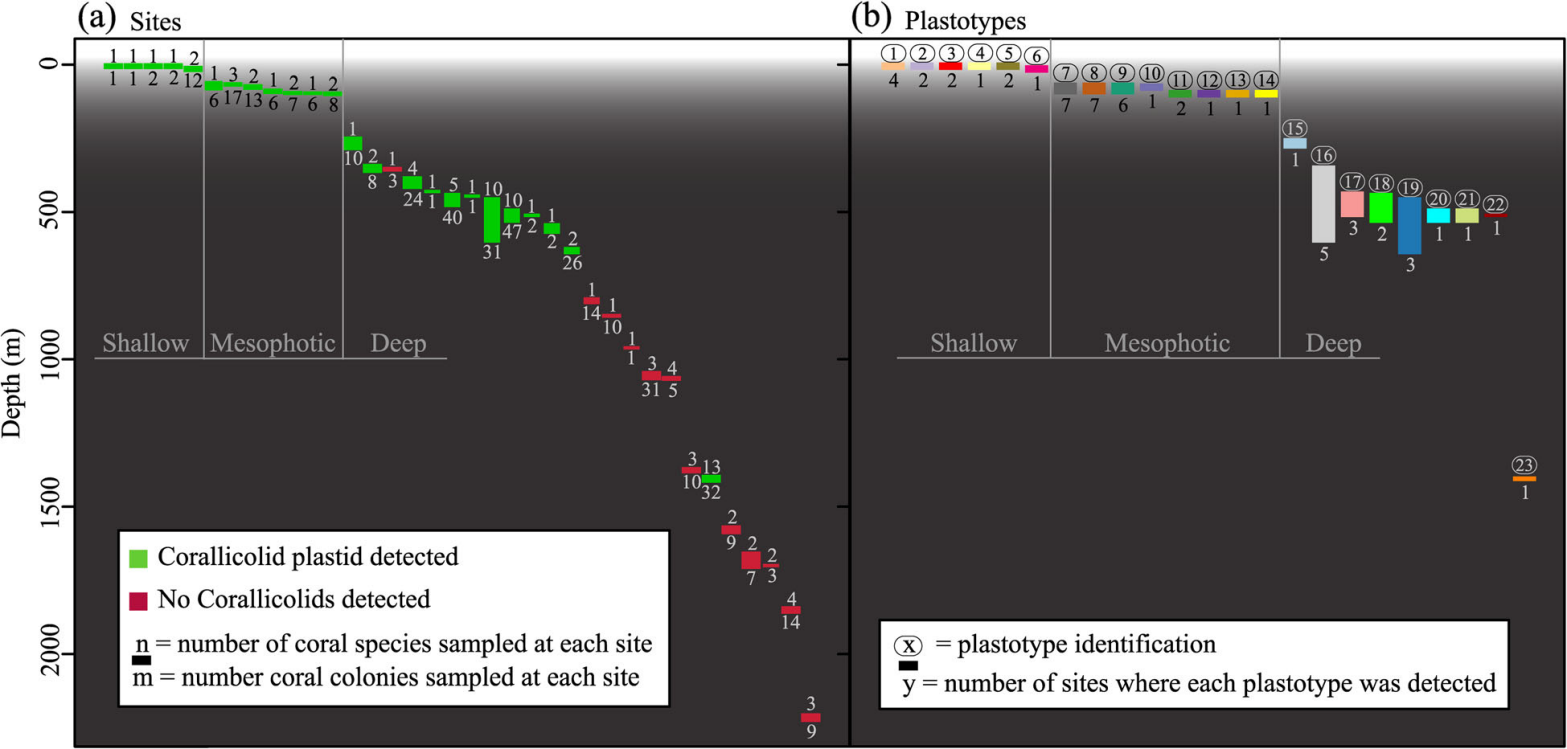
Detection of apicomplexan plastids using 16S rRNA by site across a wide depth range (**a**) and the depth ranges of each corallicolid plastotype (**b**). Numbers above the boxes in (**a**) represent the number of coral species sampled at each site while the numbers underneath denote the total number of colonies sampled. Numbers above boxes in (**b**) are plastotype identifications while the numbers underneath denote the number of sites where each plastotype was detected

In total, 25 plastotypes were identified based on the 16S rRNA gene and 23 of these corresponded to corallicolid plastids (ARL-V). Corallicolid plastotypes were detected among 14 coral species including scleractinians (five plastotypes, one coral species), antipatharians (five plastotypes, three coral species), alcyonaceans (13 plastotypes, ten coral species), and between three major depth zones: shallow (0–20 m, six plastotypes 1–6, three coral species), mesophotic (60–100 m, eight plastotypes 7–14, three coral species), and deep-sea (250–1400 m, nine plastotypes 15–23, eight coral species) (Fig. 4b). Two plastotypes (24 and 25) clustered outside of ARL-V (Fig. S2 in Additional file 1). One was found in two colonies of the mesophotic coral *Swiftia exserta* (plastotype 24), and a second was found in a single deep-sea *Stichopathes* sp*.* colony (plastotype 25).

### Depth and geographic patterns of plastid markers

**Fig. 5:**
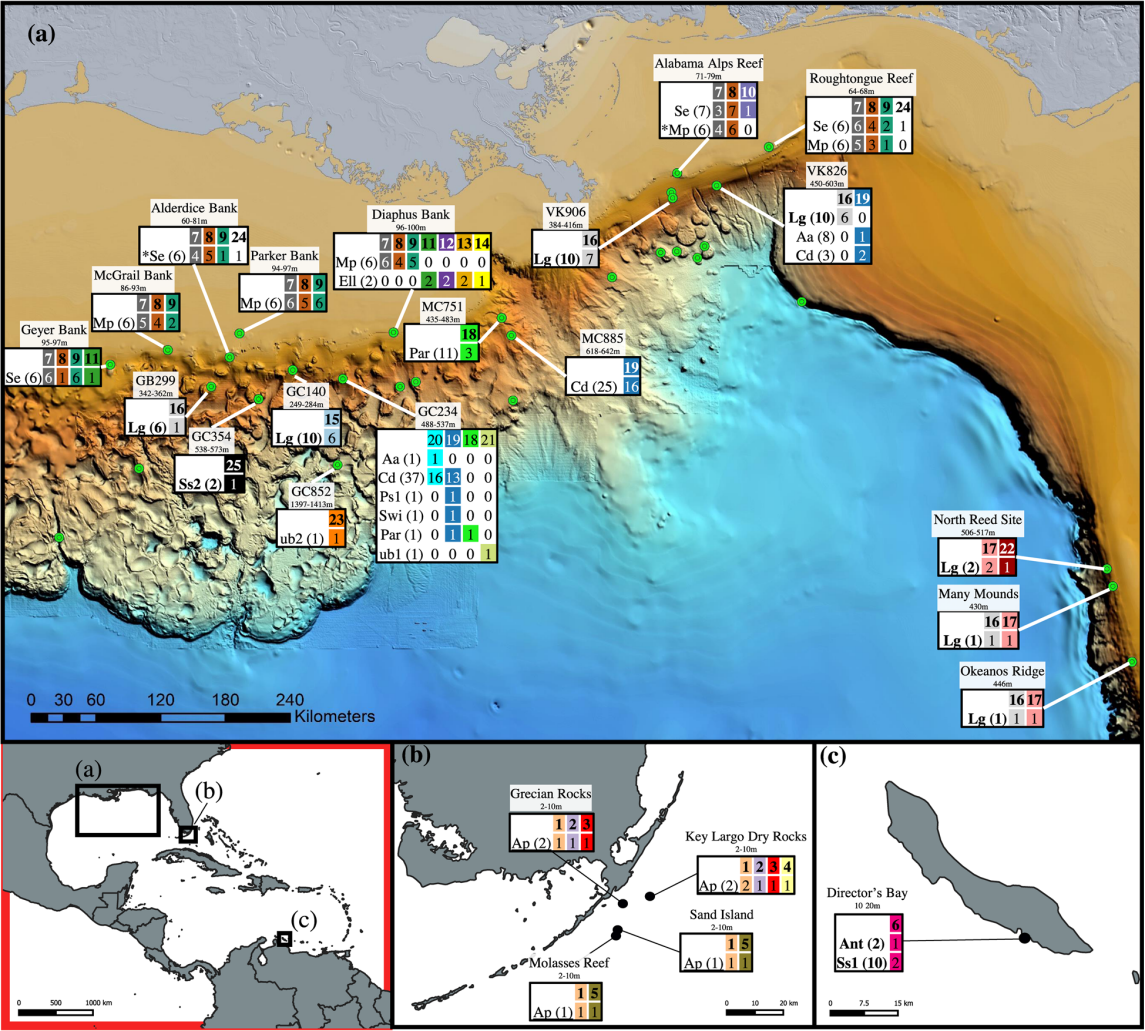
Map of coral sampling locations where corallicolid plastids were detected using 16S sequences in the northern Gulf of Mexico (**a**), Florida Keys (**b**), and Curaçao (**c**). Coral species that hosted corallicolids are listed for each site using abbreviations described in Table S2 in Additional File 1. Numbers in parentheses next to species abbreviations denote the number of colonies screened. Each 16S plastotype is denoted by a letter and unique color and/or pattern. Numbers in the tables denote the number of colonies of each species in which each plastotype was detected. Bathymetric map of the Gulf of Mexico is available from U.S. Bureau of Ocean Energy Management Gulf of Mexico Deepwater Bathymetry Grid. *For colonies that were sampled more than once, a plastotype was considered detected if it was found in at least one sample from that colony

**Fig. 6:**
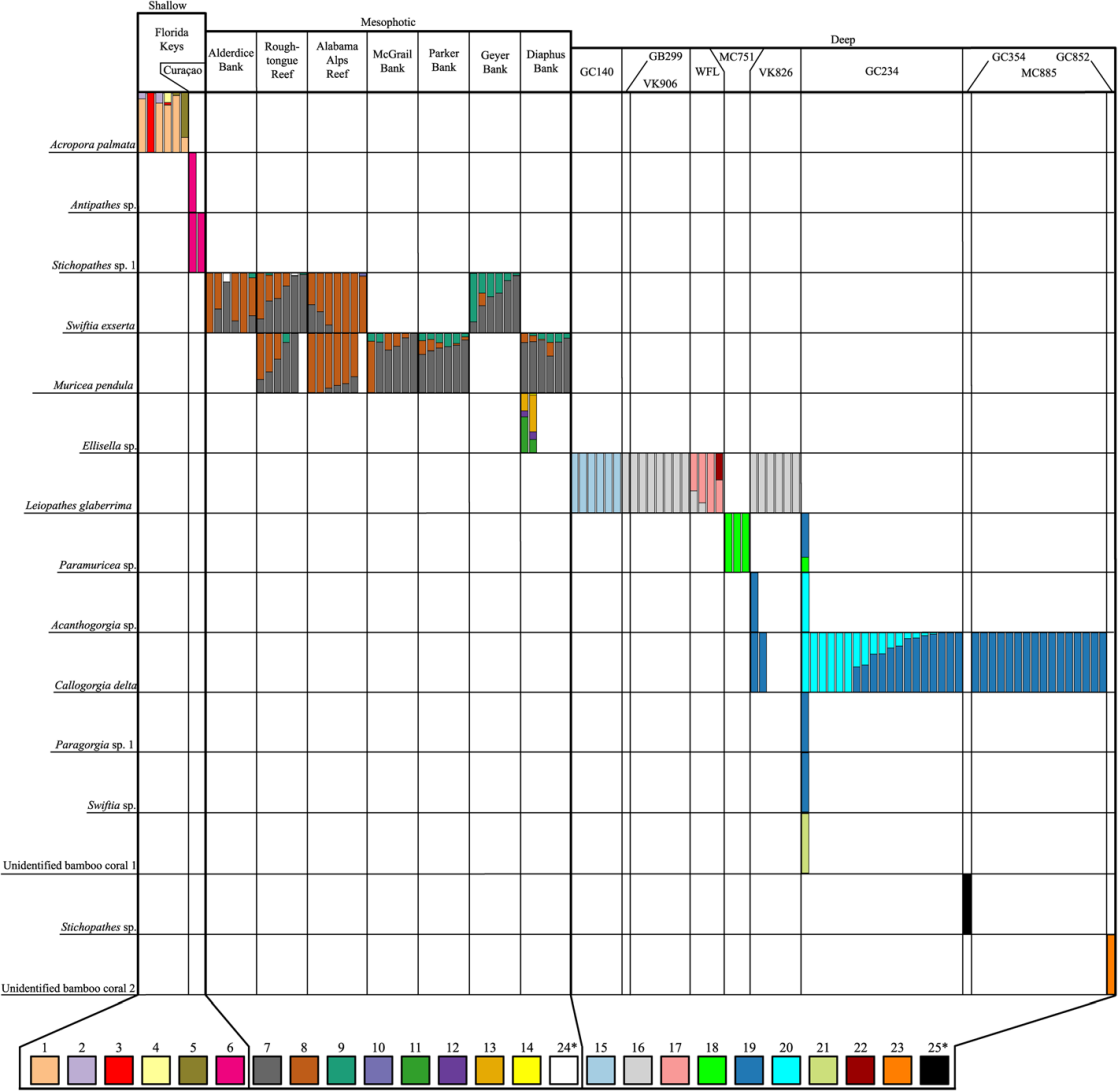
Site and host patterns in the relative abundances of plastotypes. Each bar represents the relative abundances of each plastotype among all plastid sequences found in an individual coral colony

Corallicolid plastids were detected down to 1400 m but were rare below 700 m (Fig. 4). All plastotypes were restricted to a single depth zone (shallow, mesophotic, or deep). However, some had wide geographic ranges within a depth zone (Figs. 4, 5, and 6). For instance, plastotypes 7 and 8 were present in *Swiftia exserta* and/or *Muricea pendula* at all seven mesophotic sites spanning 563 km, and plastotype 16 was found in *L*. *glaberrima* from six deep-sea sites spanning 794 km east to west (Fig. 5). While some plastotypes had wide ranges, others were not found at every site where their host corals were sampled (Fig. 6). For instance, plastotype 19 associated with *C*. *delta* colonies at sites GC234 (13 out of 37), MC885 (16/25), and VK826 (2/3), but no plastotype was detected in any *C*. *delta* colony from MC751 (*n* = 19), GC249 (*n* = 14), or GC290 (*n* = 10). Additionally, plastotype 17 was only found in *L*. *glaberrima* colonies from sites on the West Florida Slope and was absent from four other sites where *L*. *glaberrima* was sampled and other plastotypes were present in this species (Fig. 6).

### Host flexibility and specificity of plastid markers

Many pairings between coral host and apicomplexan symbiont were flexible. At least six plastotypes were detected in multiple coral species (Figs. 5 and 6). For instance, plastotypes 7, 8, and 9 were found in both *S*. *exserta* and *M*. *pendula* across their ranges. Similarly, plastotype 19 was found in five deep-sea octocorals including *C*. *delta*, *Acanthogorgia aspera*, *Paragorgia* sp. 1, *Paramuricea* sp., and *Swiftia* sp. Pairings were also flexible from the host perspective since many coral species hosted multiple plastotypes as did many individual colonies. Eight coral species hosted at least two plastotypes. Among colonies with apicomplexans, about 47% hosted multiple plastotypes, and some hosted up to four in the same sample. Despite these flexibilities, some potential patterns of specificity were observed. For instance, plastotype 18 was only detected in the genus *Paramuricea* at both MC751 and GC234 despite the presence of other coral species that hosted corallicolids at these sites. Further, every plastotype was restricted to one order of corals.

### Phylogenetic patterns among corallicolids using plastid markers

**Fig. 7:**
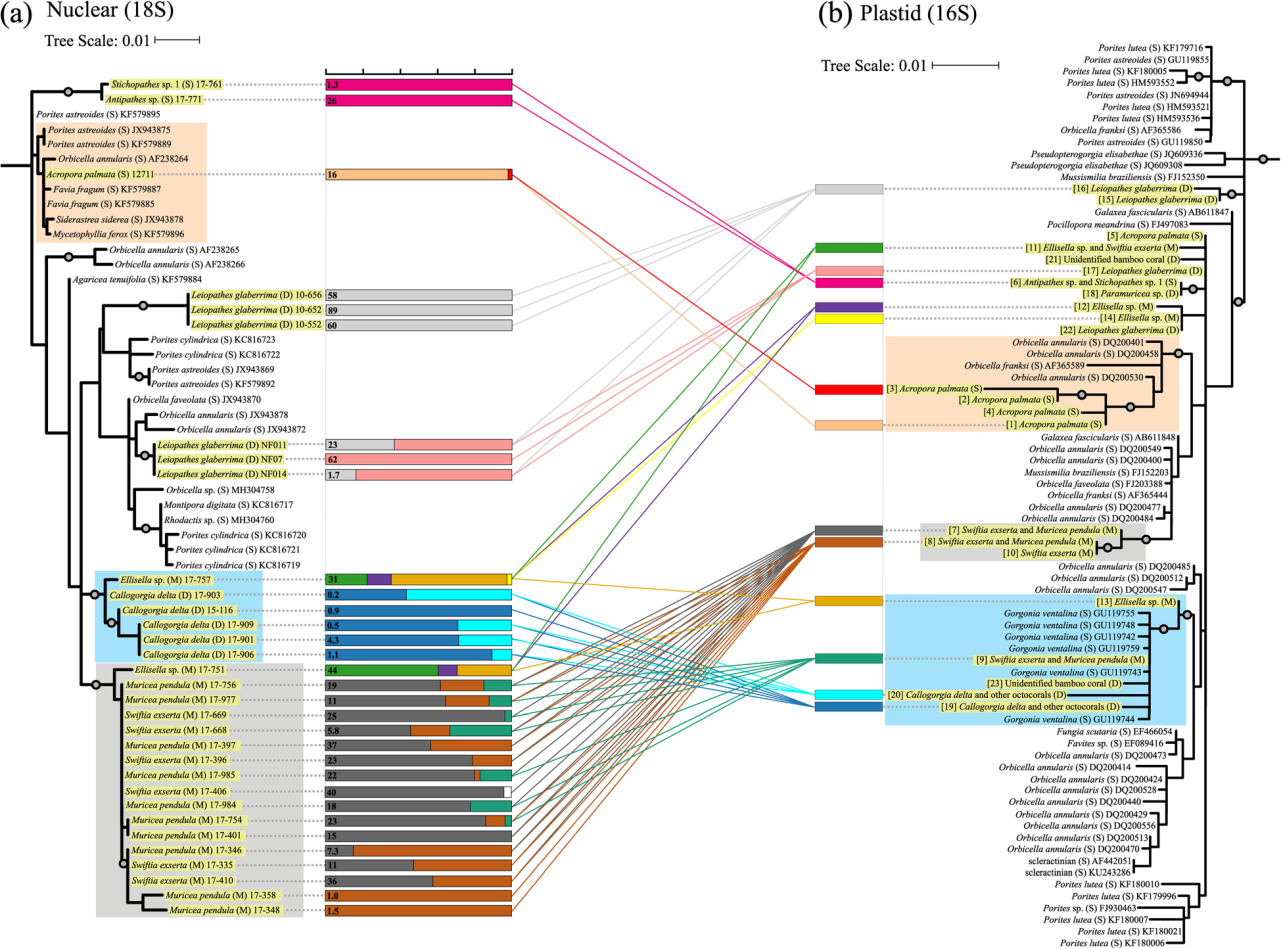
Phylogenetic relationships among corallicolids using both nuclear (**a**) and plastid (**b**) markers. Sequences are labeled by host coral species and are designated shallow-water (S), mesophotic (M), or deep-sea (D). Corallicolid plastotypes based on 16S are labeled 1–23. Sequences obtained in this study are highlighted in yellow. Circles represent nodes with greater than 95% ultrafast bootstrap support. Alongside each 18S sequence, the relative abundances of all plastotypes obtained from the same sample are displayed in barplots. For each sample, the total relative abundance of apicomplexan plastotypes among all 16S sequences is denoted on the left of each bar. Lines connect each bar to the 16S plastotypes present within it. Outgroups are not shown. See Fig. S2 in Additional File 1 for outgroups

Phylogenetic relationships among corallicolids based on plastid sequences were not strictly governed by coral host phylogeny, depth, or geography (Fig. 7). For instance, corallicolid plastid sequences obtained from octocorals did not form a single cluster and were instead dispersed among those obtained from scleractinians. Similarly, sequences from deep-sea corals did not form a single cluster. Further, plastotypes detected in the same coral colony were not always their closest relatives such as plastotypes 16 and 17 which co-occurred within some *L*. *glaberrima* colonies but had divergent sequences*.* Despite this lack of general patterns, five well-supported clusters included sequences from host coral species restricted to a single order or lower taxonomic level. Two clusters with good bootstrap support (> 95%) included only sequences obtained from octocorals. One of these clusters included octocoral hosts from shallow, mesophotic, and deep-sea habitats and included five plastotypes obtained in this study (9, 13, 19, 20, and 23). Further, two well supported clusters (> 98%) only included sequences recovered from shallow-water scleractinian corals. One of these clusters included four plastotypes found in *A*. *palmata* (plastotypes 1–4), which formed a well-supported cluster (99%) by themselves. Finally, plastotypes 15 and 16 were obtained from *L*. *glaberrima* and formed their own well-supported cluster (98%).

### Phylogenetic patterns among corallicolids using nuclear markers (18S sequences)

Thirty-two nuclear-encoded 18S sequences corresponding to genotype N were recovered from eight coral species including one scleractinian, three antipatharians, and four alcyonaceans. Like the phylogenetic relationships inferred from plastid sequences, those inferred from nuclear sequences were not strictly clustered based on coral host, depth, or geography. Despite this, several well-supported clusters included sequences obtained from a single coral species or order (Fig. 7). For instance, sequences obtained from *Leiopathes glaberrima* from Viosca Knoll sites (VK826 and VK906) formed a well-supported cluster (100%) which was distantly related to a second cluster of sequences obtained from *L*. *glaberrima* from the West Florida Slope (95%). Two other clusters were well supported (> 97%) and only included sequences obtained from octocorals. One of these clusters included sequences from *Callogorgia delta* and *Ellisella* sp. and further contained another well-supported subcluster (98%) of sequences that were obtained from *C*. *delta* only. The second octocoral cluster included sequences from the mesophotic corals *Ellisella* sp., *Swiftia exserta*, and *Muricea pendula* from all seven sites*.* Within this cluster, sequences from *S*. *exserta* and *M*. *pendula* clustered with 90% support; however, the relatively small amount of structure within this cluster did not reflect coral host species or site.

## Discussion

The newly discovered apicomplexans named “corallicolids” have been identified in diverse anthozoan hosts, yet their function is unknown. By surveying a broad diversity of anthozoans from a variety of habitats, we have obtained insight into the ecology, evolution, and potential role of this novel group of apicomplexans in corals.

### Ecology

This dataset provides some clues regarding the potential niche preferences of corallicolids and their mode of transmission. Here, we expand the known distribution of corallicolids to a depth of at least 1400 m. Many corallicolids were detected in mesophotic and deep-sea coral species demonstrating that corallicolids as a group are general anthozoan associates that are not restricted to corals in tropical shallow-water reefs. However, corallicolids were not as prevalent at sites deeper than about 700 m despite extensive sampling down to 2200 m (Fig. 4). The underlying cause of this pattern is not clear. Corallicolids may be limited to a certain depth if they have a function that utilizes light. While corallicolids do have plastids which encode some genes in chlorophyll synthesis, there is no indication of any function that involves light. Alternatively, if corallicolids rely on vector species as other apicomplexans do, their transmission may be limited in deeper water where mobile species exist at lower densities. Another possibility is that corallicolids were not present in the particular coral species that we sampled below 700 m but are present in other corals at those depths since corallicolids were not detected in all coral species at shallower depths. Comparisons to additional detection methods, coral species, and/or ocean basins will be necessary to establish the consistency of this pattern.

While corallicolids as a group had a wide range, specific plastotypes differed in niche characterized by habitat and varying host coral specificity. For instance, many apicomplexan plastotypes were widespread, but all were restricted to corals within a depth zone. The only exception was plastotype 16 which was present at very high relative abundances in *L*. *glaberrima* colonies at Viosca Knoll sites but was also detected at low abundances in samples from other depth zones and coral orders (Fig. S1 in Additional File 1). We suggest that this is likely to have resulted from contamination or sample cross-talk [39, 40] because the only samples which contained plastotype 16, other than those from *L*. *glaberrima*, were processed on a sequencing run which included 30 *L*. *glaberrima* samples. Plastotype 16 was present at very low relative abundance in these samples (including two of four Powersoil kit extraction blanks and one of four PCR negative controls) and many had fewer reads that passed quality control and likely had low DNA concentrations and were more susceptible to contamination or sample cross-talk. Further, no plastotype 16 was found in any coral except *L*. *glaberrima* processed on other sequencing runs. Finally, 21 colonies had replicates on both sequencing runs. In ten colonies, plastotype 16 was detected in the replicate on the first sequencing run but was not detected in any replicate on the second sequencing run. Plastotype 16 was not detected in either the first or second sequencing run in the remaining 11 colonies.

The restriction of all other plastotypes to a single depth zone may, in part, reflect the fact that all coral species sampled were also restricted to a single depth zone. However, some plastotypes were found in divergent alcyonaceans over a 500-km span but were not found in other alcyonacean species as close as 30 km away in deeper waters. In addition to habitat preference, all corallicolids were limited by host phylogeny. No corallicolid was detected with confidence in coral species which belonged to multiple orders. Some plastotypes seemed restricted to a single coral species or genus, such as plastotype 18, which was only found in *Paramuricea* sp. at two separate sites despite the presence of other octocorals nearby. Others, however, were flexible, such as the corallicolids which were detected in both *Swiftia exserta* and *Muricea pendula* which belong to separate families [41]. This presence of the same plastotypes in these two coral species is unlikely to be due to contamination (see Supplement for explanation). Host specificity of individual corallicolids has not yet been reported for this group. Previous work, however, has only found patterns in the community of plastid sequences in three coral species [28].

The data further suggest that some corallicolids may be horizontally transmitted. First, specific corallicolids were detected in divergent coral species indicating that these corallicolids may disperse between colonies independent of their host corals’ larvae. Second, some corallicolids were not detected at all sites where their host coral species were sampled. For example, we failed to detect corallicolids in *C*. *delta* at MC751 using both 16S surveys and metagenomes despite detecting corallicolids in around 50% of *C*. *delta* colonies within three other sites. Third, the distribution of some corallicolids did not reflect their host population structure. For instance, plastotype 17 was only found in *Leiopathes glaberrima* on the West Florida Slope, and we failed to detect it in colonies at GC140 and GB299, even though these host populations are connected via gene flow [36]. It is possible that additional corallicolids may be present at sites where they were not detected because they were below our threshold of detection. However, this seems unlikely given the number of colonies screened.

### Evolution

As previously reported [28], mitochondrial sequences of corallicolids clustered within apicomplexa alongside coccidians. However, our analysis of plastid sequences also placed corallicolids within apicomplexans while Kwong et al. placed them basal to apicomplexans. This may be due to our selection of only the five longest protein coding genes while Kwong used 19 genes and examined multiple tree building methods. This discrepancy may be resolved as a greater diversity of apicomplexan relatives are analyzed.

Several clades of corallicolids were associated with coral hosts from a single order or lower taxonomic level (Fig. 7) which suggests the possibility of coevolution between corallicolids and their coral hosts within these groups. However, the relationships between these clades did not reflect the coral phylogeny, depth zone, or geography. Multiple clusters of corallicolids grouped with other corallicolids that associated with corals from different orders and depth zones. Some corallicolids even infected multiple coral species while corallicolids from divergent clades infected the same coral species and even the same individual. This suggests that corallicolid lineages have transitioned between depth zones and coral order multiple times and thus exhibit flexibility over evolutionary timescales.

The lack of strong large-scale phylogenetic correlations with host, habitat, or geography may be due to the relatively sparse sampling of the tremendous anthozoan diversity and their wide geographic ranges. The inclusion of corallicolids from additional coral species, depths, and locations may reveal undetected phylogenetic patterns. Taxonomic and phylogenetic descriptions of another coral-associated alveolate group, dinoflagellates in the family Symbiodiniaceae, was stymied for decades by similar challenges because the family is composed of multiple speciose genera with varying levels of host specificity [15]. Some species of zooxanthellae show high host specificity while others are host generalists [42, 43]. Conversely, some corals only associate with one species of zooxanthellae while other corals are more flexible [42].

Several patterns appeared in both nuclear and plastid phylogenies including clusters of sequences from the same or closely related host coral species (Fig. 7). For instance, a clade including sequences from both *C*. *delta* and *Ellisella* sp. appeared in both nuclear and plastid phylogenies (Fig. 7, blue boxes). Similarly, a second clade including sequences from *Swiftia exserta* and *Muricea pendula* was present in both phylogenies (Fig. 7, gray boxes). However, the nuclear and plastid phylogenies based on single marker genes of corallicolids showed limited phylogenetic congruence. The shared clusters mentioned above did not cluster in the same positions relative to each other when comparing trees (Fig. 7). For instance, the 18S sequences obtained from shallow-water antipatharians and the cluster including our sequence from *Acropora palmata* were the most basal sequences yet their associated plastid sequences were placed in more derived positions. These incongruencies may reflect the low support values for deeper level branches and the limited power of these sequences to resolve these relationships.

Definitive alignments between the nuclear and plastid phylogenies are also limited by the difficulty in assigning plastotypes to nuclear sequences because the variation of plastid sequences associated with a single nuclear lineage is unknown and may vary at multiple levels (Fig. 1). First, the two copies of 16S may differ within a plastid genome. Second, corallicolids may be heteroplasmic if individual cells harbor plastids which exhibit sequence variation [44]. Third, corallicolids with similar nuclear sequences may harbor divergent plastids. Finally, there may be hidden diversity in nuclear sequences that was not detected and is associated with some of the divergent 16S sequences. For instance, all corallicolid 18S sequences obtained from *S*. *exserta* and *M*. *pendula* formed a single cluster yet the plastotypes detected in the same samples belonged to two divergent clades. Plastotypes 7 and 8 may be associated with the cluster of 18S sequences detected in these corals but plastotype 9, which was present at lower relative abundances, may be associated with an 18S lineage that was not detected.

### Role of the symbiont

Contrary to early speculation [31], we conclude that corallicolid plastids are not likely to be involved in photosynthesis. Kwong et al. [28] reached a similar conclusion because the plastid genome of corallicolids did not encode photosystem genes; however, they did not rule out their presence in the nuclear genome. Our survey provides further evidence against photosynthesis since many corallicolids were found in deep-sea coral species at depths below the photic zone. Further, LIPOR genes and *acsF* were confirmed in the plastids of corallicolids that associate with corals at depths greater than 400 m.

While a role involving photosynthesis can be confidently ruled out for corallicolids, we cannot rule out a parasitic lifestyle like that of their apicomplexan relatives. Previous speculation has discounted parasitism since corallicolids were not associated with a recognizable pathology in their coral hosts. However, even the well-established parasitic coccidian apicomplexan, *Toxoplasma gondii*, appears asymptomatic in most human infections. It only results in more severe pathologies in a small percentage of immunocompetent humans [45–48]. Corallicolids may be similar parasites in corals, with a minor impact on host fitness under normal circumstances but increasing in abundance and impact on the host when the corals are stressed. This is consistent with the observation that apicomplexans increased in abundance in corals afflicted with “white plague” disease [49]. Further, the original work that described *Gemmocystis cylindrus* reported patchy bleaching and tissue necrosis in some infected corals [20]. However, without appropriate controlled experiments, it is difficult to determine the role of these apicomplexans in corals.

In addition to the lifestyle of corallicolids, the function of their LIPOR genes and *acsF* remains uncertain. These genes may still be producing chlorophyll for an unknown purpose. This seems unlikely since Kwong et al. [28] reported no autofluorescence from corallicolids while autofluoresence was observed in co-occurring photosynthetic Symbiodiniaceae in the shallow-water corallimorph they examined. Another possibility is that the LIPOR gene products may have evolved a different function altogether. This gene family has a history of dramatic functional shifts since the genes of the LIPOR complex share an evolutionary origin with *nif* genes which compose a nitrogen-fixing enzyme complex [50]. In corallicolids, the LIPOR complex may perform a different role that uses its Fe-S centers as active sites, such as the synthesis of isoprenoids or lipoic acid. These compounds are synthesized by other enzymes with Fe-S centers in the apicoplasts of apicomplexans and are essential for their survival in their hosts [51].

Whatever its function, we hypothesize that the LIPOR enzyme in corallicolids is sensitive to oxygen concentrations and may be related to a low oxygen niche for several reasons: First, oxygen impedes the LIPOR enzyme complex in other organisms by binding its Fe-S centers [52–54]. Second, corallicolids were detected in the mesenteries in the gastric cavity of polyps which can be a hypoxic to anoxic environment in corals [55]. Third, some of the closest relatives to corallicolids based on 18S sequences were recovered from anoxic marine sediment from Greenland [19]. Fourth, certain life stages of other apicomplexans appear to be adapted to a low oxygen niche by relying primarily on fermentation [56, 57]. Finally, some enzymes that conduct essential functions of plastids in other apicomplexans, such as isoprenoid biosynthesis, are impeded by oxygen [58, 59]. Altogether, this suggests that corallicolids may occupy a microoxic niche in corals and share many characteristics with parasitic apicomplexans.

## Conclusions

Here, we provide the first foray into a systematic survey of a novel anthozoan associate: corallicolids. We show that corallicolids are more widespread and diverse than previously thought by acquiring and analyzing samples from underrepresented taxa and habitats. We found that corallicolids form a diverse, apicomplexan clade with members that appear to be limited to coral hosts within a taxonomic order and within broad depth zones. We detected several clades of corallicolids that associated with closely related coral hosts. However, some corallicolid lineages show signs of transitioning between coral hosts and habitats over evolutionary time scales. Finally, the presence of LIPOR genes encoded in the plastids of apicomplexans associated with deep-sea corals suggests that the plastid’s role is not photosynthesis and is perhaps related to a low oxygen environment within coral polyps. These genes may represent another toolkit that has been repurposed from a role in photosynthesis to a new role as the ancestor of apicomplexans evolved towards a parasitic lifestyle.

## Methods

### Collections

Four hundred twenty-one coral colonies from 42 coral morphospecies including scleractinians (hard corals), antipatharians (black corals), zoantharians, and alcyonaceans were sampled from 33 sites in the Gulf of Mexico, Curaçao, and the Florida Keys ranging in depth from 2 to 2224 m. Thirty-two of those morphospecies were collected from 21 deep-sea sites in the Northern Gulf of Mexico ranging in depth from 249 to 2224 m and spanning 897 km east to west (Fig. 5, Table S1 in Additional File 1). The deep-sea collection sites are named for the Bureau of Ocean Energy Management lease block in which they are found, for example MC751 is lease-block 751 in the Mississippi Canyon lease area. Five coral species were collected from seven mesophotic sites ranging in depth from 60 to 100 m. The mesophotic and deep-sea corals were collected on eight research cruises between 2009 and 2017 with remotely operated vehicles (ROVs) onboard the vessels NOAAS *Ronald H Brown* (2009 and 2010, ROV Jason II), R/V *Atlantis* (2014, HOV Alvin), E/V *Nautilus* (2015, ROV Hercules), DSV *Ocean Inspector* (2016, ROV Global Explorer), MSV *Ocean Intervention II* (2017, ROV Global Explorer), DSV *Ocean Project* (2017, ROV Comanche), and NOAAS *Nancy Foster* (2017, ROV Odysseus). Each ROV dive was restricted to one site and thus depth changed little within a dive (10–20 m). Branches of mesophotic and deep-sea corals were sampled using specially designed coral cutters on the manipulator arm of the ROV and stored in separate compartments of a temperature-insulated biobox or separated within quivers using specially designed rubber stoppers. Upon recovery of the ROV, corals were transferred to cold seawater (< 10 °C), and subsamples were flash frozen or stored in 90% ethanol within 4 h. Frozen samples were stored at − 80 °C and ethanol samples at − 20 °C before DNA extraction. We attempted to flash freeze all samples but since liquid nitrogen was limited on some cruises, we preserved some samples in 90% ethanol instead including *Muricea pendula* from Diaphus Bank and McGrail Bank; *Ellisella* sp. from Diaphus Bank; *Swiftia exserta* from Geyer Bank; *Callogorgia delta* from MC751, MC885, and GC234 in 2015 but not other years; *Leiopathes glaberrima* from sites on the West Florida slope; and a single *Swiftia pallida* colony from GC852.

Three coral species were collected from shallow-water sites. Six colonies of *Acropora palmata* in total were sampled from four sites in the Florida Keys in November 2014 (Table S1 in Additional File 1). Coral fragments were removed using a hammer and chisel, snap frozen at the surface, and stored at − 80 °C until extraction. Two *Antipathes* sp. and ten *Stichopathes* sp. colonies were collected from Director’s Bay in Curaçao in March 2017 from 10 to 20 m depth. Coral fragments were removed using bone cutters and kept in individual Ziploc bags with seawater for transportation to CARMABI Research Station where they were preserved in 90% ethanol and stored at − 20 °C until DNA extraction.

### Metagenomes

To construct metagenomic libraries, DNA was extracted from 12 *Leiopathes glaberrima* colonies using a Powersoil DNA extraction kit (Qiagen, Hilden, Germany) and from eight *Callogorgia delta* and four *Paramuricea* sp. B3 colonies using a DNA/RNA Allprep kit (QIAGEN, Hilden, Germany) with RLT+ lysis buffer with β-mercaptoethanol. DNA/RNA Allprep kits were used on these species because it produced extracts with higher DNA concentrations and less degradation for these species. Whole tissue including skeleton was homogenized using bead-beating (Powersoil kit) or a tissue homogenizer (Allprep kit). All library preparation was conducted at the Max Planck Genome Center in Cologne, Germany and sequenced on an Illumina Hiseq2500 platform with 100 bp paired-end reads for all *L*. *glaberrima* colonies and 150 bp paired-end reads for both *C*. *delta* and *P.* sp. B3 metagenomes. Four of these *Leiopathes glaberrima* libraries were subsequently sequenced deeper using 250 bp paired-end reads.

Twenty-one publicly available *Acropora palmata* metagenomes were used under the sequence read archive accession numbers SRR7235979-SRR7235980, SRR7235982, SRR7235983, SRR7235985-SRR7235988, SRR7236001, SRR7236003, SRR7236007-SRR7236012, SRR7236015, and SRR7236017-SRR7236020. These samples originate from four distinct areas spanning the entire range of this species in the Caribbean: Florida Keys (*n* = 6), US Virgin Islands (*n* = 5), Belize (*n* = 5), and Curaçao (*n* = 5) [60].

Metagenomes were quality filtered by trimming reads to a quality score of greater than 2 using bbduk (ver 37.52) and screened for ARL-V using phyloFlash (ver3.3) [61]. The *Leiopathes glaberrima* library with the highest ARL-V coverage was assembled using Megahit (ver1.0.5) [62] under default parameters and a k-max of 141. A single circular contig of 6300 bp was identified as the mitochondrial genome based on its sequence similarity to coccidian apicomplexan mitochondrial genes (NCBI BLASTn) [63]. A draft plastid assembly was constructed using a contig containing the ARL-V 16S rRNA gene and two other contigs linked in the De Bruijn assembly graph using Bandage ver0.8.1 [64]. These three contigs were combined into one scaffold manually because they had significant overlap. A final putative plastid contig was identified by coverage and GC content and contained sequences similar to apicoplasts (NCBI BLASTn). The mitogenome and draft plastid genome were annotated using GeSeq within the Chlorobox web server [65] using tRNAscan-SE [66]. The *Eimeria tenella* mitogenome (HQ702484) and a corallicolid mitogenome and plastid genome (MH320093, MH324845) were used as references.

All metagenomes were screened for the presence of corallicolids with bbmap (Bushnell, B. Available from: https://sourceforge.net/projects/bbmap/) by mapping reads to the corallicolid 18S, mitogenome, and plastid genome from *Leiopathes glaberrima*. Corallicolid mitogenomes and plastid genomes were considered detected in a library if reads mapped with greater than 90% identity and 96% coverage for 250 bp and 150 bp libraries and 98% identity and coverage for 100 bp libraries. Apicomplexan nuclear 18S was considered detected if reads mapped with greater than 95% identity and 96% coverage for 150 bp and 250 bp reads. No reads from any 100 bp library could be confidently classified as corallicolid 18S.

### 16S rRNA metabarcoding

All coral samples were screened for the presence of ARL-V by 16S rRNA metabarcoding. DNA was extracted from deep-sea corals, mesophotic corals, and corals from Curaçao using Powersoil DNA extraction kits. Four extraction blanks were processed alongside coral samples using Powersoil DNA extraction kits. Some samples were processed using other extraction kits. DNeasy kits (QIAGEN, Hilden, Germany) were used for *Acropora palmata* samples (*n* = 36) because these samples were originally used in a separate project [67] and four colonies of *Callogorgia americana* were processed using DNA/RNA Allprep kits since these were older samples and we wanted to increase the chance of obtaining high-quality DNA extracts. For all samples, whole coral tissue including skeleton was homogenized using bead-beating for Powersoil DNA extraction kits or a tissue homogenizer for DNeasy and DNA/RNA Allprep kits. To compare extraction procedures, five colonies each of *Paramuricea biscaya*, *Callogorgia delta*, *Swiftia exserta*, and *Muricea pendula* were extracted using both a DNA/RNA Allprep kit using RLT+ lysis buffer with β-mercaptoethanol and a Powersoil DNA extraction kit. Finally, replicate extractions using the same kit were performed on some colonies allowing comparisons of the detection rate of apicomplexan plastids. Six replicate extractions were performed on all six *Acropora palmata* colonies. Also, 2–4 replicate extractions were performed on each of six colonies of *Callogorgia delta.*

The V1-V2 region of the 16S rRNA gene was amplified using the general bacterial primers, 27F and 355R, which were appended with Illumina CS1 and CS2 Fluidigm adapter sequences [68]. Polymerase chain reactions of 20 μL were conducted using 0.1 U/μL Gotaq (Promega, Madison, WI), 1× Gotaq buffer (Promega, Madison, WI), 0.25 mM dNTPs (Bioline, Alvinston, Canada), 2.5 mM MgCl_2_, and 0.25 μM of each primer. Initial denaturation lasted 5 min at 95 °C. This was followed by 30 cycles of 30 s at 95°, 1 min at 51 °C, and 1 min at 72 °C. Final extension lasted 7 min at 72 °C. Successful amplification was checked on a 1% agarose gel. The DNA services facility at the University of Illinois Chicago prepared libraries and sequenced all samples on two separate runs on an Illumina MiSeq platform. Four negative PCR controls were processed alongside other samples using sterile water instead of DNA extract and sequenced on the first run.

Amplicon libraries of 16S genes were analyzed using QIIME2 version 2017.11 [69] using default parameters unless otherwise stated. Paired reads were joined using vsearch and quality filtered using “quality-filter q-score-joined.” Chimeras were detected and removed, sub-operational taxonomic units (sOTU’s) were constructed, and amplicons were trimmed to 300 bp using “deblur denoise-16S.” These sOTU’s represent a lower taxonomic level than OTU’s based on clustering and sequence similarity thresholds. These sOTU’s were classified as plastid sequences if their representative sequence had greater than 90% similarity to the plastid 16S rRNA gene assembled from the *L*. *glaberrima* metagenomes. These are hereafter referred to as plastotypes.

All plastotypes were considered present in a sample if at least one read was detected. However, plastotype 16 was only considered present in samples on the first sequencing run if it composed 15% or higher relative abundance. This is because plastotype 16 from *Leiopathes glaberrima* likely contaminated other samples since many other samples on the first sequencing run contained plastotype 16 at very low abundance including negative controls. See “Discussion” section for further explanation.

### 18S rRNA amplicon sequencing

An 860 bp section of the nuclear-encoded 18S rRNA gene was sequenced from selected samples using a PCR primer pair designed to amplify apicomplexan 18S in the presence of coral and *Symbiodinium* spp. [18 N-F2 5′-TAGGAATCTAAACCTCTTCCA-3′; 18 N-R1 5′-CAGGAACAAGGGTTCCCGACC-3′] [21]. PCR was performed in 50 μL reactions using 3.0 mM MgCl_2,_ 0.5 μM of each primer, 0.5 mM dNTPs, 0.15 U/μL taq polymerase (Bioline, Alvinston, Canada), and 1× NH_4_ buffer (Bioline, Alvinston, Canada). Thermocycler conditions followed the touchdown program designed by Kirk et al. [29, 30]. Amplification was verified using 2% agarose gels in 1× TAE buffer, and bands at approximately 850 bp were excised. DNA was extracted from gels using a gel extraction kit (QIAGEN, Hilden, Germany), then Sanger sequenced at the Penn State Genomics Core facility. 18S sequences were analyzed, trimmed, and assembled using default parameters in CodonCode aligner (ver 3.7.1).

### Phylogenetic trees

Our phylogenetic analyses included publicly available sequences from an additional 16 species of shallow-water scleractinians and 2 species of shallow-water octocorals. The 18S and 16S sequences were aligned in Geneious 11.0.3 using a MUSCLE alignment (ver 3.8.425) [70]. Maximum likelihood trees were constructed using iqtree (ver1.6.10) [71], model selection was performed using ModelFinder [72], and 1000 bootstrap replicates were performed using UFboot2 [73]. A Tamura-Nei model [74] and a Hasegawa, Kishino, and Yano model [75] were selected as base substitution models for 18S sequences and 16S sequences, respectively. Both models utilized empirical base frequencies and a 2-category free rate model for rate heterogeneity across sites.

Trees from concatenated single gene alignments were constructed to identify the phylogenetic position of the corallicolid mitogenome and plastid genome using three genes (*cytb*, *cox1*, and *cox3*) and five genes (*clpC*, *rpoB*, *rpoC1*, *rpoC2*, and *tufA*), respectively. Amino acid sequences were aligned and concatenated in Geneious, an amino acid substitution model test was performed in MEGA X, and a maximum likelihood tree was constructed using the Le Gascuel model with a discrete Gamma distribution with five parameters in MEGA X.

## Supplementary information

**Additional file 1.** Supplemental Information. Additional text, figures, and tables with sampling locations, species abbreviations, description of the potential contamination of plastotype 16, and a comparison of extraction procedures on apicomplexan detection.

**Additional file 2.** 16S rRNA amplicon screening data. This dataset includes the read counts for all plastotype sequences for all samples.

**Additional file 3.** Metagenomic screening data. This dataset includes all mapping results of screening the metagenomes for apicomplexans.

## Data Availability

The datasets generated by this study were deposited in Genbank under accession numbers MK996254-MK996278 (plastotype sequences), MN022428-MN022459 (apicomplexan 18S sequences), MN078134 (mitochondrial genome), and MN586853 and MN586854 (plastid genome). Raw metagenomic and amplicon sequence data generated in this study is available on the National Center for Biotechnology Information (NCBI) Sequence Read Archive (SRA) under BioProject numbers PRJNA565265 and PRJNA574146 and BioSample IDs SAMN12824622–4713, SAMN12824722–4964, SAMN12825090–5207, SAMN12825209–5269, and SAMN12856788—SAMN12856811 https://www.ncbi.nlm.nih.gov/sra. These data are also publicly available through the Gulf of Mexico Research Initiative Information & Data Cooperative (GRIIDC) at https://data.gulfresearchinitiative.org (doi: <10.7266/RYMQTDQ9> and R4.x268.000:0126). The genomic sequence data from *Acropora palmata* that was analyzed in this study is available in the NCBI SRA database under accession numbers SRR7235979–80, 82–83, 85–88, SRR7236001–03, 07–12, 15, and 17–20.
