# Supplementary material for: Deep-sea corals provide new insight into the ecology, evolution, and the role of plastids in widespread apicomplexan symbionts of anthozoans
Source: Microbiome. 2020 Mar 12;8:34. doi: 10.1186/s40168-020-00798-w (PMC7068898; doi:10.1186/s40168-020-00798-w)
Supplement: Supplementary file 2 — Additional file 1. Supplemental Information. Additional text, figures, and tables with sampling locations, species abbreviations, description of the potential contamination of plastotype 16, and a comparison of extraction procedures on apicomplexan detection. [file 40168_2020_798_MOESM2_ESM.pdf]

Supplementary Information for

Deep-sea corals provide new insight into the ecology, evolution, and the role of plastids in widespread apicomplexan symbionts of anthozoans.

Samuel A. Vohsen, Kaitlin E. Anderson, Andrea M. Gade, Harald R. Gruber-Vodicka, Richard P. Dannenberg, Eslam O. Osman, Nicole Dubilier, Charles R. Fisher, Iliana B. Baums

Corresponding author: Samuel Vohsen
Email: sav146@psu.edu

This PDF file includes:

Supplementary text
Figures S1 and S2
Tables S1 to S5

Supplementary Information Text

Comparing extraction procedures and detection rates

Apicomplexan plastids were detected in a higher percentage of colonies using the DNA/RNA AllPrep kit (Qiagen, Hilden, Germany) compared to the DNeasy PowerSoil kit (Qiagen). For both *S. exserta* and *M. pendula,* apicomplexans were detected in all 5 colonies using the DNA/RNA AllPrep kit but were only detected in 3 of those same colonies using the DNeasy PowerSoil kit (Table S3). Further, plastotypes 7, 8, 9 and 24 were all individually detected in more colonies using the DNA/RNA AllPrep kit. The detection rate was the same between kits, however for *C. delta* in which plastotypes U and T were detected in all 5 colonies using both kits and *P. biscaya* in which no apicomplexans were detected in any colony regardless of extraction kit used.

Apicomplexan plastids were not detected in all replicates from the same colony. For instance, plastotype 1 was found in five *A. palmata* colonies but its detection ranged from 1-6 replicates (Table S4). In addition, in a colony of *C. delta*, 2 replicates contained both plastotypes 19 and 20 while a third contained neither. In all 5 other *C. delta* colonies with multiple replicates, no apicomplexan plastids were detected in any replicates.

Factors that affect the detection rate of apicomplexan plastids

Apicomplexan plastid occurrence has been reported as high as 100% among coral colonies; however, there are several factors that affect this detection rate [27, 28]. First, the extraction procedure affected the rate of detection. In general, the DNA/RNA AllPrep kit was more likely to detect apicomplexans compared to the more commonly used DNeasy PowerSoil kit. Second, apicomplexans were more likely to be detected if more replicates were processed per coral colony. The failure to detect apicomplexans in some replicates may be due not only to non-biological factors such as extraction efficiency or sequencing depth but also to biological factors such as the relative abundance of apicomplexans or a non-uniform distribution across the coral colony. Altogether, this may suggest that a higher percentage of individual coral colonies host apicomplexans than previously reported and comparisons of the detection rate between species is difficult.

Contamination is not likely why the same plastotypes appear in *Swiftia* and *Muricea*

We believe it is unlikely that *Swiftia exserta* and *Muricea pendula* share plastotypes due to contamination for various reasons. First, these corals were collected in separate containers. Second, three other coral species were collected at sites where *Muricea* and/or *Swiftia* were collected however none of these other corals harbored these

plastotypes. In fact, *Ellisella* hosted its own set of plastotypes. Finally, we collected only *Swiftia* or only *Muricea* at five separate sites and these corals still harbored plastotypes 7, 8, and 9 (often within a single colony).

Contamination of plastotype 16

Plastotype 16 was present in roughly 1/3 of the samples in the first sequencing run. In the second sequencing run, this plastotype was restricted to *Leiopathes glaberrima* samples in which it composed over 80% of the microbial community in some samples. In almost all samples that were not *Leiopathes glaberrima,* plastotype 16 composed less than 5% of the microbial community (Fig. S1). The only exception was a sample with fewer reads that passed quality control and thus it was likely a low-quality sample that was probably more susceptible to contamination. Plastotype 16 was only considered present in samples from the first sequencing run if it composed more than 5% of the microbial community except for the one sample with few reads that passed quality control.

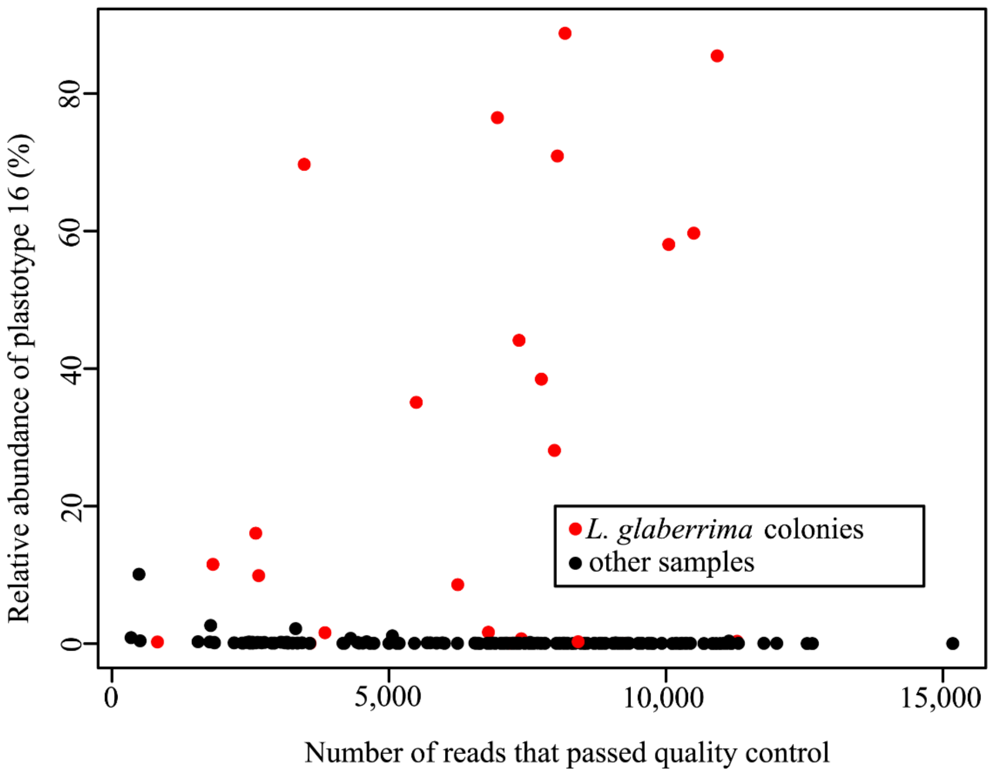

Fig. S1. Contamination of plastotype 16 is suggested comparing the number of reads that passed quality control and the relative abundance of plastotype 16.

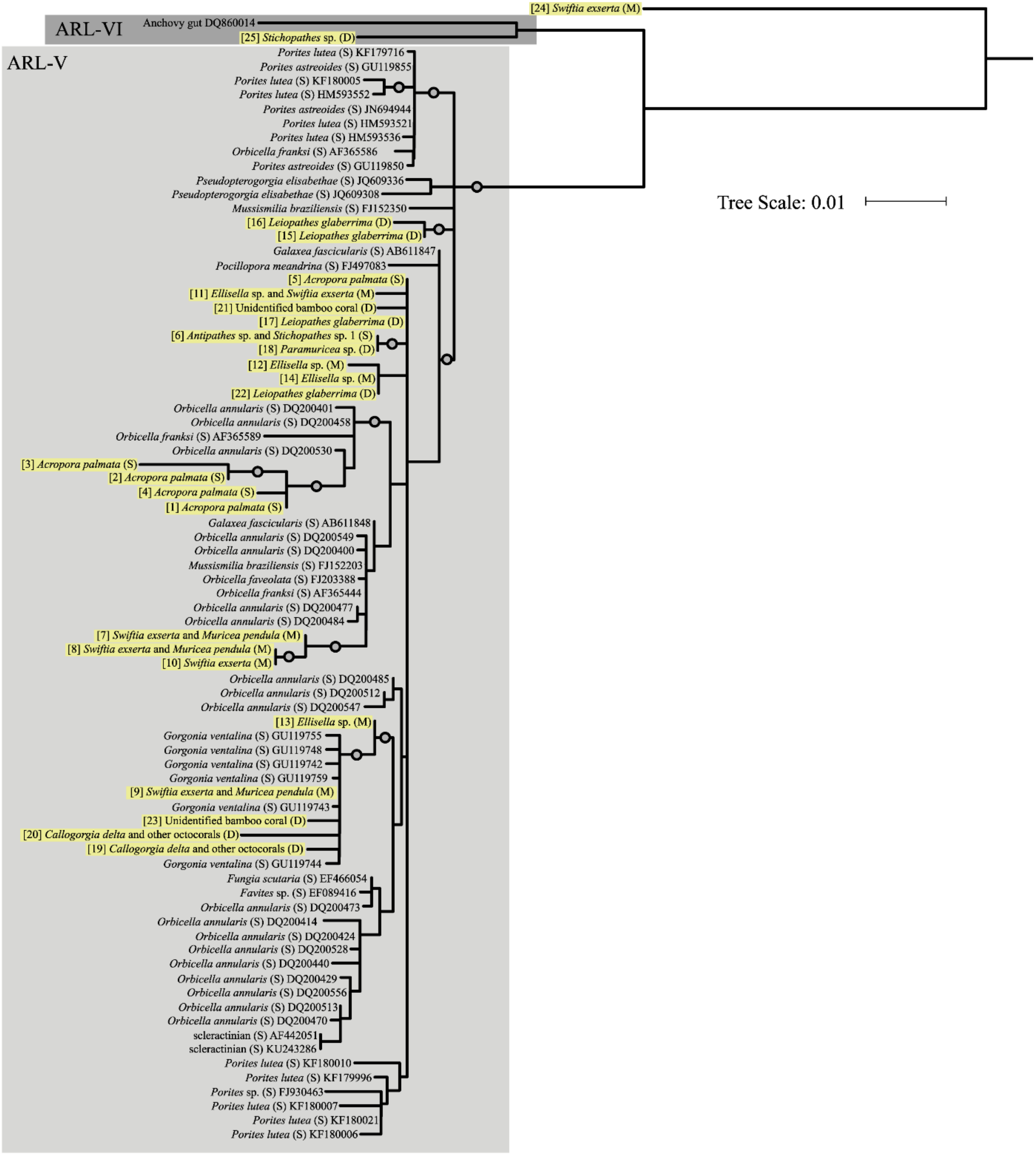

Fig. S2. Maximum Likelihood phylogenetic tree including all plastotypes with a HKY + F + 2 model. Nodes are denoted with gray dots if they had greater than 95% support among 1000 ultrafast bootstrap iterations. ARL = apicomplexan-related lineage.

Table S1. Sampling locations and coral species sampled. Scleractinian species are underlined, antipatharian species are indicated in bold, zoantharians are italicized, and alcyonaceans are in regular text. Numbers in parentheses denote the number of colonies of each species sampled at each site. Sites and species abbreviations highlighted in yellow indicate where corallicolids were detected in at least some individuals from that site or species. A key to species abbreviations is in Table S2.

	Site name	Lat.	Long.	depth (m)	Species collected
Shallow	Grecian Rocks	25.111	-80.369	2-10	Ap (2)
	Molasses Reef	25.010	-80.373	2-10	Ap (1)
	Sand Island	25.018	-80.369	2-10	Ap (1)
	Key Largo Dry Rocks	25.123	-80.297	2-10	Ap (2)
	Director's Bay	12.066	-68.861	10-20	**Ant (2), Ss1 (10)**
Mesophotic	Alderdice Bank	28.079	-91.982	60-81	Se (6)
	Roughtongue Reef	29.440	-87.576	64-68	Se (6), Mp (6), uo2 (5)
	Alabama Alps Reef	29.250	-88.339	71-79	Se (7), Mp (6)
	McGrail Bank	27.957	-92.579	86-93	Mp (6)
	Parker Bank	27.904	-92.065	94-97	Mp (6), Cg (1)
	Geyer Bank	27.849	-93.058	95-97	Se (6)
	Diaphus Bank	28.086	-90.700	96-100	Mp (6), Ell (2)
Deep	GC140	27.810	-91.539	249-284	**Lg (10)**
	GB299	27.689	-92.218	342-362	**Lg (6)**, Ca (2)
	VK862	29.109	-88.387	352-357	Ca (3)
	VK906	29.069	-88.377	384-416	**Lg (10)**, Par (7), Mh (3), Lp (4)
	Many Mounds	26.206	-84.712	430	**Lg (1)**
	MC751	28.193	-89.800	435-483	Cd (19), Par (11), Mh (4), Ps2 (1), Lp (5)
	Okeanos Ridge	25.640	-84.552	446	**Lg (1)**
	VK826	29.159	-88.010	450-603	**Lg (10), Bs1 (1), Sib (1)**, Aa (8), Cd (3), Ci (1), Swi (1), Ps2 (1), Lp (4), *uz1 (1)*
	GC234	27.746	-91.122	488-537	Cd (37), Aa (1), Mh (2), Ps1 (1), Par (1), ub1 (1), Swi (1), uo1 (1), ucc (1), Lp (1)
	North Reed Site	26.337	-84.760	506-517	**Lg (2)**
	GC354	27.597	-91.823	538-573	**Ss2 (2)**
	MC885	28.064	-89.718	618-642	Cd (25), Lp (1)
	GC249	27.724	-90.514	789-812	Cd (14)
	GC290	27.689	-90.646	851-853	Cd (10)

	MC462	28.491	-88.881	961	Chr (1)
	AT357	27.587	-89.705	1045-1064	PB3 (24), Mo (4), *uz2 (3)*
	GB903	27.083	-92.819	1062-1066	Pr (2), PB3 (1), Aa (1), Chr (1)
	MC294	28.672	-88.477	1371-1381	Pb (8), Pr (1), Sp (1)
	GC852	27.110	-91.166	1397-1413	**Bs2 (1)**, Pb (11), PB3 (3), Ps (1), Sp (3), ub2 (1), ub3 (1), Chr (1), Cor (1), Nar (1), Is (1), Er (4), Mo (3)
	MC297	28.680	-88.342	1569-1587	**Bs2 (2)**, Pb (7)
	KC405	26.571	-93.483	1657-1705	Pb (6), Sp (1)
	MC258	28.719	-88.111	1699	**Bs2 (1), Ss3 (2)**
	MC344	28.634	-88.170	1843-1857	**Bs2 (3), Ss3 (3),** Pb (7), Cr (1)
	DC673	28.313	-87.302	2206-2224	**Bs2 (2), Ss2 (1)**, Pb (6)

Table S2. Species and morphospecies abbreviations used in Table S1 and Figure 1. Species in which apicomplexans were detected are highlighted.

Aa	*Acanthogorgia aspera*	Pb	*Paramuricea biscaya*
**Ant**	*Antipathes* sp.	PB3	*Paramuricea* sp. type B3
Ap	*Acropora palmata*	Par	*Paramuricea* sp.
**Bs1**	*Bathypathes* sp. 1	Pr	*Paragorgia regalis*
**Bs2**	*Bathypathes* sp. 2	Ps1	*Paragorgia* sp. 1
Ca	*Callogorgia americana*	Ps2	*Paragorgia* sp. 2
Cd	*Callogorgia delta*	Se	*Swiftia exserta*
Cg	*Callogorgia gracilis*	**Sib**	*Sibopathes* sp.
Chr	*Chrysogorgia* sp.	Sp	*Swiftia pallida*
Ci	*Chelidonisis aurantiaca*	**Ss1**	*Stichopathes* sp. 1
Cor	*Corallium* sp.	**Ss2**	*Stichopathes* sp. 2
Cr	*Clavularia rudis*	**Ss3**	*Stichopathes* sp. 3
Ell	*Ellisella* sp.	Swi	*Swiftia* sp.
Er	*Enallopsammia rostrata*	ub1	unidentified bamboo coral 1
Is	*Iridogorgia splendens*	ub2	unidentified bamboo coral 2
**Lg**	*Leiopathes glaberrima*	ub3	unidentified bamboo coral 3
Lp	*Lophelia pertusa*	ucc	unidentified cup coral
Mh	*Muriceides hirta*	uo1	unidentified octocoral 1
Mo	*Madrepora oculata*	uo2	unidentified octocoral 2
Mp	*Muricea pendula*	*uz1*	zoanthid on *Chelidonisis*
Nar	*Narella* sp.	*uz2*	unidentified parazoanthid on PB3

Table S3. Number of colonies in which apicomplexan plastids were detected using the DNA/RNA AllPrep kit VS. the DNeasy PowerSoil kit.

Species	Plastotype				At least one Plastotype
	7	8	9	24	
Swiftia exserta	3 vs 1	4 vs 3	1 vs 0	1 vs 0	5 vs 3
Muricea pendula	3 vs 2	5 vs 3	0 vs 0	0 vs 0	5 vs 3

Table S4. The numbers of replicates from the same colony in which distinct corallicolid plastotypes were detected.

			Plastotype						
Species	Colony	Replicates	1	2	3	4	4	19	20
Acropora palmata	1	6	6	1	0	0	0		
	2	6	5	1	0	0	0		
	3	6	5	0	0	0	1		
	4	6	5	0	3	0	0		
	5	6	1	0	0	0	3		
	6	6	0	0	2	0	0		
Callogorgia delta	1	3						2	2
